# PACAP ameliorates hepatic metabolism and inflammation through up‐regulating FAIM in obesity

**DOI:** 10.1111/jcmm.14453

**Published:** 2019-07-03

**Authors:** Xing Xiao, Pei Qiu, Hui‐Zhen Gong, Xue‐Ming Chen, Yan Sun, An Hong, Yi Ma

**Affiliations:** ^1^ Department of Cellular Biology, Institute of Biomedicine, National engineering research center of genetic Medicine, Key laboratory of Bioengineering Medicine of Guangdong Province Jinan University Guangzhou Guangdong People's Republic of China

**Keywords:** FAIM, inflammation, liver, metabolism, obesity, PACAP

## Abstract

Obesity is considered a chronic inflammatory disease, the inflammatory factors, such as interleukin 6 (IL‐6), monocyte chemoattractant protein 1 (MCP‐1) and small inducible cytokine A5 (RANTES), are elevated in obese individuals. Pituitary adenylate cyclase‐activating polypeptide (PACAP) suppresses anti‐inflammatory cytokines and ameliorates glucose and lipid metabolism. Our previous study showed that Fas apoptosis inhibitory molecule (FAIM) is a new mediator of Akt2 signalling, increases the insulin signalling pathway and lipid metabolism. In this study, we found that PACAP promoted the expression of FAIM protein in a human hepatocyte cell line (L02). Overexpression of FAIM with lentivirus suppressed the expression of the inflammatory factor interleukin 6 (IL‐6), monocyte chemoattractant protein 1 (MCP‐1) and tumour necrosis factor alpha (TNF‐α). Following treatment of obese mice with FAIM or PACAP for 2 weeks, inflammation was alleviated and the bodyweight and blood glucose levels were decreased. Overexpression of FAIM down‐regulated the expression of adipogenesis proteins, including SREBP1, SCD1, FAS, SREBP2 and HMGCR, and up‐regulated glycogen synthesis proteins, including Akt2 (Ser474) phosphorylation, GLUT2 and GSK‐3β, in the liver of obese mice. However, down‐regulation of FAIM with shRNA promotes obesity. Altogether, our data identified that FAIM mediates the function of PACAP in anti‐inflammation, glucose regulation and lipid metabolism in obese liver.

**Highlights**

- PACAP up‐regulates FAIM through the PAC1 receptor.
- FAIM inhibits the hepatic inflammation.
- PACAP‐FAIM pathway modulates adipose infiltration in liver.
- FAIM downregulates SREBP signalling and restrains lipogenesis in liver.

## 1 INTRODUCTION

Obesity is a global health disorder in the modern world. Since 1980, the number of obese individuals has more than doubled according to the World Health Organization.^1^ In 2014, more than 1.9 billion adults were overweight, and over 600 million of those people were obese.^2^ Obesity is a highly complex multifaceted disease and one of the strongest risk factors for the development of type 2 diabetes mellitus (T2DM).^3^ Obesity is also associated with an increased risk for other metabolic, cardiovascular and chronic inflammatory diseases, such as dyslipidaemia, non‐alcoholic fatty liver disease (NAFLD), hypertension, coronary heart disease and stroke.^4^ With the increasing number of obese humans, the deepening of obesity research and influence of chronic inflammation on obesity, increasing attention has been given to the effect of inflammatory cytokines on obesity.^5^ In rodents, knockout models of innate immune factors or anti‐inflammatory treatments have been demonstrated to dampen inflammation‐driven insulin resistance.^6^ However, the successful use of anti‐inflammatory agents to ameliorate insulin resistance in obese patients with diabetes in clinical trials is yet to be fully confirmed. Several avenues currently under investigation hold promise for the use of anti‐inflammatories to treat obesity‐driven T2DM. Immunoneutralization of TNF with the monoclonal antibody etanercept significantly reduces the fasting glucose levels in obese patients and lowers systemic inflammatory markers in obese patients with diabetes.^7^, ^8^

PACAP has been found to play an important role in the regulation of metabolism.^9^ However, the role of the PACAP and its high‐affinity receptor PAC1 in appetite and energy homeostasis has not been clearly elucidated yet. PAC1‐deficient mice have been previously described to have pulmonary hypertension and heart failure.^10^ Intraperitoneally injected PACAP1‐38 and PACAP1‐27 in WT mice induced a dose‐dependent decrease in cumulative food intake during the dark phase of feeding. PACAP/PAC1 suppression of appetite and feeding behaviour is linked to the inhibition of active ghrelin release and regulation of GLP‐1, insulin and leptin hormone secretion.^11^ PACAP down‐regulates the pro‐inflammatory cytokines, such as monocyte chemoattractant protein‐1 (MCP‐1) and interleukin‐6, which had been induced by the activation of toll‐like receptor (TLR) with lipopolysaccharide.^12^ These findings suggested that PACAP could be a possible treatment option for diabetic nephropathy through the use of anti‐inflammation effects on glomerular podocytes.

Akt2 is the predominant isoform in insulin‐responsive tissues such as the liver, muscle and adipose tissue.^13^ Fas apoptosis inhibitory molecule (FAIM) is a critical mediator of Akt2 activation^14^ and was initially cloned from antigen‐activated B cells and was shown to protect them from activation‐induced cell death.^15^ FAIM is ubiquitously expressed and conserved in evolution, although it bears no homology to any known protein.^16^ Alternate splicing of FAIM generates a short (FAIM‐S) isoform and a long (FAIM‐L) isoform.^15^ Mechanistically, FAIM modulates the localization of Akt2 to lipid rafts during its activation.^14^ Because Akt2 acts as a nodal point in insulin signalling and maintains glucose and lipid homoeostasis.^17^ It is conceivable that FAIM might play a role in insulin signalling and maintenance of metabolic homoeostasis. In our previous study, we found in the FAIM‐KO liver, lipogenesis was elevated, as indicated by increased fatty acid synthesis and SREBP‐1 and SREBP‐2 activation. Notably, protein expression of insulin receptor beta was markedly reduced in the insulin target organs of FAIM‐KO mice. Akt2 phosphorylation was also lower in FAIM‐KO liver and adipose tissue than in WT controls. In addition, phosphorylation of insulin receptor substrate‐1 (IRS1) and Akt2 in response to insulin treatment in isolated FAIM‐KO hepatocytes was also markedly attenuated. FAIM is a novel regulator of insulin signalling and plays an essential role in energy homoeostasis.^18^

In order to expose the relationship between the two metabolism regulators (PACAP and FAIM), we knocked down FAIM with lentivirus, then the effects of PACAP‐FAIM pathway on the body weight, inflammation, metabolism and steatosis were analysed. The results proved that FAIM could mediate the function of PACAP on glucose and lipid metabolism. These findings provided a novel pathway and therapy target for obesity or diabetes individuals.

## 2 MATERIALS AND METHODS

### 2.1 Methods

Human recombinant PACAP1‐38 was obtained from Tocris Bioscience (R&D Systems, Minnesota, USA). Lipopolysaccharide (LPS) was obtained from Sigma Aldrich (Guangzhou, China). The VPAC1‐selective inhibitor PG99‐465, VPAC2‐selective inhibitor PG99‐465 and PAC1‐selective inhibitor MAX.D.4 were custom synthesized by GL BioChem, Shanghai, People's Republic of China. The serum levels of TNF‐α, IL‐6, RANTES and MCP‐1 were measured using ELISA kits (Ray Biotech, Atlanta, USA). The levels in obese human and healthy controls are shown in Table S1. The serum levels of glucose, free fatty acid, triglyceride and total cholesterol were measured using the Glucose Colorimetric Assay Kit (Cayman Chemical, Ann Arbor, MI, USA), Free Fatty Acid Quantification Colorimetric/Fluorometric Kit (Bio Vision, Milpitas, USA), Triglyceride Colorimetric Assay Kit (Cayman Chemical, St. Louis, USA) and Cholesterol Quantitation Kit (Sigma‐Aldrich, St. Louis, USA). Penicillin and streptomycin were obtained from Invitrogen (Carlsbad, CA). Human peripheral blood leucocyte collection kit was obtained from Omega Bio‐Tek (Guangzhou, China). The fasting peripheral blood was collected from simple obese and healthy subjects (The first affiliated hospital of Jinan university). The protein expression of FAIM and PACAP in peripheral blood leucocyte were analysed.

### 2.2 Cell culture

L02 cells were cultured in RPMI‐1640 (Gibco, Carlsbad, CA) with 10% foetal bovine serum (FBS, Gibco), penicillin (100 units mL^−1^) and streptomycin (100 µg mL^−1^) in a humidified atmosphere containing 5% CO_2_ at the temperature of 37℃. L02 cells were preincubated for 24 hours at 37°C with different gradient concentrations of PACAP (100 µmol/L, 10 µmol/L, 1 µmol/L, 100 nmol/L, 10 nmol/L or 1 nmol/L). For VPAC2, VPAC1 and PAC1 receptor blocking experiments, the selective inhibitors PG99‐465, PG97‐269 and MMAX.D.4 were used. L02 cells were pre‐incubated for 24 hours at 37°C with different groups (1 µmol/L PACAP, 1 µmol/L PACAP + 1 µmol/L MAX.D.4, 1 µmol/L PACAP + 1 µmol/L PG99‐465, 1 µmol/L PACAP + 1 µmol/L PG97‐269). Alpha mouse liver 12 (AML12 and LO2) cells, a normal mouse liver cell line, was purchased from the Type Culture Collection of the Chinese Academy of Sciences (Shanghai, China) and were cultured in DMEM/F12 (Gibco, Carlsbad, CA,USA) supplemented with 10% foetal bovine serum (FBS), ITS liquid media supplement (Sigma, St. Louis, MO, USA), dexamethasone (40 ng/mL) and 100 µg/mL each of penicillin and streptomycin (Invitrogen) in 5% CO_2_ at 37°C. The low glucose DMEM/F12 was a 1:1 mixture of no glucose DMEM (Gibco) and Ham's F‐12 medium (Gibco).

### 2.3 Generation and administration of the lentivirus gene expression vectors

The following lentivirus gene expression vectors were used: LV‐FAIM: Vector Name: pLV[Exp]‐EGFP:T2A:Puro‐EF1A>mFaim [NM_011810.3], Size: 9914 bp, Vector ID: VB170418‐1013zrf; LV‐shFAIM1: Vector Name: pLV[shRNA]‐EGFP:T2A:Puro‐U6>mFaim[shRNA#1], Size: 8347 bp, Vector ID: VB170418‐1121zvf; LV‐shFAIM2: Vector Name: pLV[shRNA]‐EGFP:T2A:Puro‐U6>mFaim[shRNA#4], Size: 8347 bp, Vector ID: VB170418‐1123sbj; LV‐shFAIM3: Vector Name: pLV[shRNA]‐EGFP:T2A:Puro‐U6>mFaim[shRNA#5], Size: 8347 bp, Vector ID: VB170420‐1085qvk. The lentivirus gene expression vectors were diluted in phosphate‐buffered saline at a dose of 10^7^ plaque‐forming units (PFU) per well in 12‐well plates or through tail vein injection using 10^9^ PFU per mouse. The lentivirus gene expression vectors were obtained from Cyagen Biosciences Inc (Guangzhou, China).

### 2.4 Animal care and treatment

Six‐week‐old male C57BL/6J wild‐type mice were obtained from Guangdong Animal Centre (Guangdong, China). Forty wild‐type mice were fed with a 60% high‐fat (Trophic Animal Feed High‐Tech Co., Ltd. Nantong, China) diet for 10 weeks, and each of five obese mice were injected with PACAP or LV‐FAIM or LV‐shFAIM for 14 days. PACAP1‐38 was administered by intraperitoneal injection at a concentration of 100 nmol/L in 200 µL of saline into mice every other day for 14 days. The mice were fasted overnight before they were injected. Another two groups of overnight‐fasted mice were injected with LV‐FAIM or LV‐shFAIM. At last a part of LV‐shFAIM group mice were injection at a concentration of 100 nmol/L in 200 µL of saline every other day for 14 days (n = 5). Plasma samples were obtained from whole‐blood collection, and a cocktail of protease inhibitors containing protease inhibitor cocktail tablets with EDTA (Roche, Indianapolis, IN), aprotinin (Pittsburgh, PA), and dipeptidyl peptidase IV (DPP‐IV) inhibitor (Millipore, Billerica, MA) was added.

### 2.5 RT‐qPCR

To determine the mRNA expression of IL‐6, MCP‐1, TNF‐α and FAIM, qRT‐PCR analysis was performed. Total RNA was extracted from cells by TRIzol® (Invitrogen) and was treated with DNase I (Invitrogen). cDNA was synthesized from 1 μg of total RNA using random primers with the ReverTra Ace qPCR RT Kit (Takara Bio, Guangzhou, Japan). The genes MCP‐1, IL‐6, TNF‐α and FAIM were chosen for real‐time PCR using the Power SYBR Green PCR Master Mix (Takara Bio, Guangzhou, Japan); their PCR primer sequences are listed in Figure S2, and GAPDH was used as the internal reference. The 2^−ΔΔCt^ method was used to quantify the relative expression levels of each group.

### 2.6 Western blot analysis and antibodies

Cells after different treatments were collected and then Western blot analysis was performed as described previously. Next, the primary antibodies were incubated at 4°C overnight, and the bands were visualized by chemiluminescence, imaged using a Chemi Doc XRS and analysed using Image Lab (both from Bio‐Rad, Guangzhou, China). The following primary antibodies from Cell Signaling Technology or Abcam (Union Biotech, Guangzhou, China) were used for immunoblotting: anti‐FAIM, anti‐GLUT2, anti‐GSK‐3β, anti‐SREBP‐1, anti‐SREBP‐2, anti‐FAS, anti‐Akt2, anti‐pAkt2 (Ser474) and anti‐HMGCR.

### 2.7 Histopathological examination

The liver and abdominal adipose tissue obtained from each animal was fixed in a 10% formalin solution, processed according to routine protocol and embedded in paraffin blocks. Sections (10‐μm thick) were obtained and stained with haematoxylin and eosin (H&E), and frozen liver sections were stained with Oil Red O, followed by examination of the slides under a light microscope.

### 2.8 Measurement of the levels of liver triglycerides and liver total cholesterol

Triglycerides and total cholesterol were determined using a triglyceride kit and a cholesterol kit (Beyotime Biotechnology, Shanghai, China), respectively. These assays were performed according to the manufacturer's instructions, and the levels in each group are shown in Figure S3.

### 2.9 Accumulative glucose consumption and the insulin sensitivity assay

The glucose concentration in the culture medium was detected using the Glucose Assay Kit (Sigma). The accumulative glucose consumption was calculated using the initial concentration minus the glucose concentration at different time points. In addition, the insulin sensitivity was measured by determining the ability of glucose consumption in the presence of 100 nmol/L insulin.

### 2.10 Statistical analysis

All data are expressed as the means ± SEM. Significant differences were assessed either by two‐tailed Student's *t* test or by one‐way ANOVA, followed by the Student‐Newman‐Keuls test for most of the results.

## 3 RESULTS

### 3.1 FAIM and PACAP levels are lower in obese individuals

**Figure 1:**
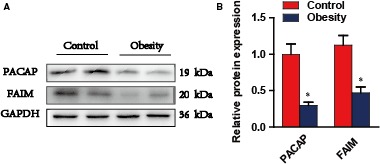
PACAP, FAIM expression and serum parameters in obese humans. A and B, Protein expression of FAIM and PACAP in blood leucocytes of obese humans (obesity, n = 2) and healthy controls (control, n = 2, *P < 0.01, obese humans versus healthy controls). Data are presented as mean ± SEM

We recruited 40 obese humans (20 females and 20 males; age = 40±18 years; body mass index (BMI) = 29.76 ± 2.28 kg/m^2^) and 20 healthy controls (10 females and 10 males; age = 22±5 years; BMI = 24.67 ± 1.8 kg/m^2^) and then detected the serum levels of pro‐inflammatory factors and lipid parameters. The results showed that the pro‐inflammatory cytokine levels in the obese group (IL‐6, MCP‐1, TNF‐α and RANTES) were significantly higher than those in the healthy control group (Figure S1A). The serum levels of TC, TG, LDL‐C and FPG in the obese group were increased by 1.23, 2.18, 1.29 and 1.58‐fold respectively, compared with those in the healthy control group. However, the serum levels of HDL‐C in the obese group were lower than the healthy control group (Figure S1B). We used the human blood leucocyte collection kit to collect leucocytes. More remarkably, the protein levels of FAIM and PACAP in leucocytes were significantly lower in the obese group than in the healthy control group (*P < 0.01) (Figure 1A and 1).

### 3.2 PACAP up‐regulates FAIM through the PAC1 receptor

**Figure 2:**
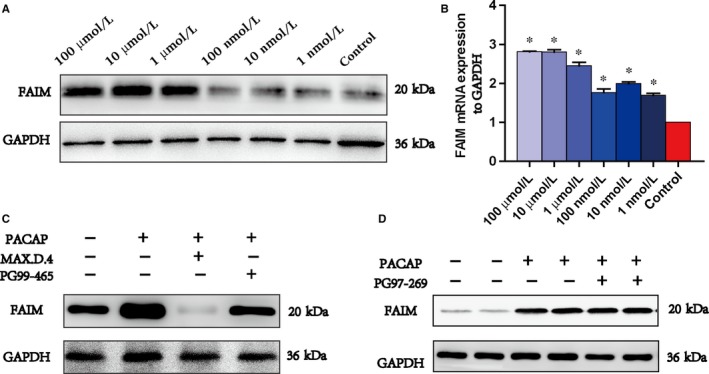
PACAP up‐regulates FAIM through the PAC1 receptor. A and B, Protein (A) and mRNA (B) expression of FAIM with different concentrations of PACAP (100 μmol/L, 10 μmol/L, 1 μmol/L, 100 nmol/L, 10 nmol/L or 1 nmol/L). *P < 0.01, PACAP‐treated groups versus control group. *P < 0.01, treated groups versus control group, #*P* > 0.05, 1 μmol/L PACAP + 1 μmol/L PG99‐465 group versus control group. C and D, Protein expression of FAIM with PACAP and inhibitors of PACAP receptors (1 μmol/L PACAP, 1 μmol/L PACAP + 1 μmol/L MAX.D.4, 1 μmol/L PACAP + 1 μmol/L PG99‐465, 1 μmol/L PACAP + 1 μmol/L PG97‐269)

To evaluate the relationship between PACAP and FAIM, we chose the human normal hepatocyte (L02 cell line) and found that PACAP could induce the protein expression of FAIM in a dose‐dependent manner (Figure 2A and 2). The mRNAs of the three receptors of PACAP (VPAC1, VPAC2 and PAC1) were all expressed in L02 cells (Figure S4A).

When the cells were treated with 1 µmol/L PG99‐465 (VPAC1 inhibitor) or 1 µmol/L PG97‐269 (VPAC2 inhibitor), the protein expression of FAIM was not influenced, while treatment with 1 µmol/L MAX.D.4 9 (PAC1 inhibitor) resulted in the protein expression of FAIM being almost restrained (Figure 2C and 2). These data indicated that PACAP could up‐regulate FAIM through the PAC1 receptor, and the similar results were obtained in AML12 cell (Figure S4B).

### 3.3 Overexpression and knockdown of FAIM in AML12 cells

To disclose the function of the PACAP‐FAIM pathway in hepatocytes, we overexpressed or knocked down FAIM in hepatocytes. First, we incubated a lentivirus expressing FAIM (LV‐FAIM) into the mouse hepatic cell line (AML12), resulting in increased mRNA and protein abundance of FAIM (Figure S5A). Second, we used a short hairpin RNA (shRNA) directed against FAIM (LV‐shFAIM), resulting in decreased mRNA and protein abundance of FAIM (Figure S5B). Third, the effects of lentivirus on FAIM expression were investigated. Lentivirus expressed green fluorescent protein (Figure S5C), but the LV‐Control had no significant influence on FAIM expression compared with the no‐lentivirus control group (FIGURE S5A and S5B). In the FAIM overexpression group (LV‐FAIM), the FAIM level was increased by 15‐fold compared with that in the control group (Figure S5A and S5D). However, in the short hairpin RNA group (LV‐shFAIM), the mRNA and protein levels of FAIM were barely expressed (Figure S5B and D). Thus, the overexpression and knockdown of FAIM in the AML12 cells were successfully constructed.

### 3.4 FAIM alleviates hepatic inflammation and glucose metabolism

**Figure 3:**
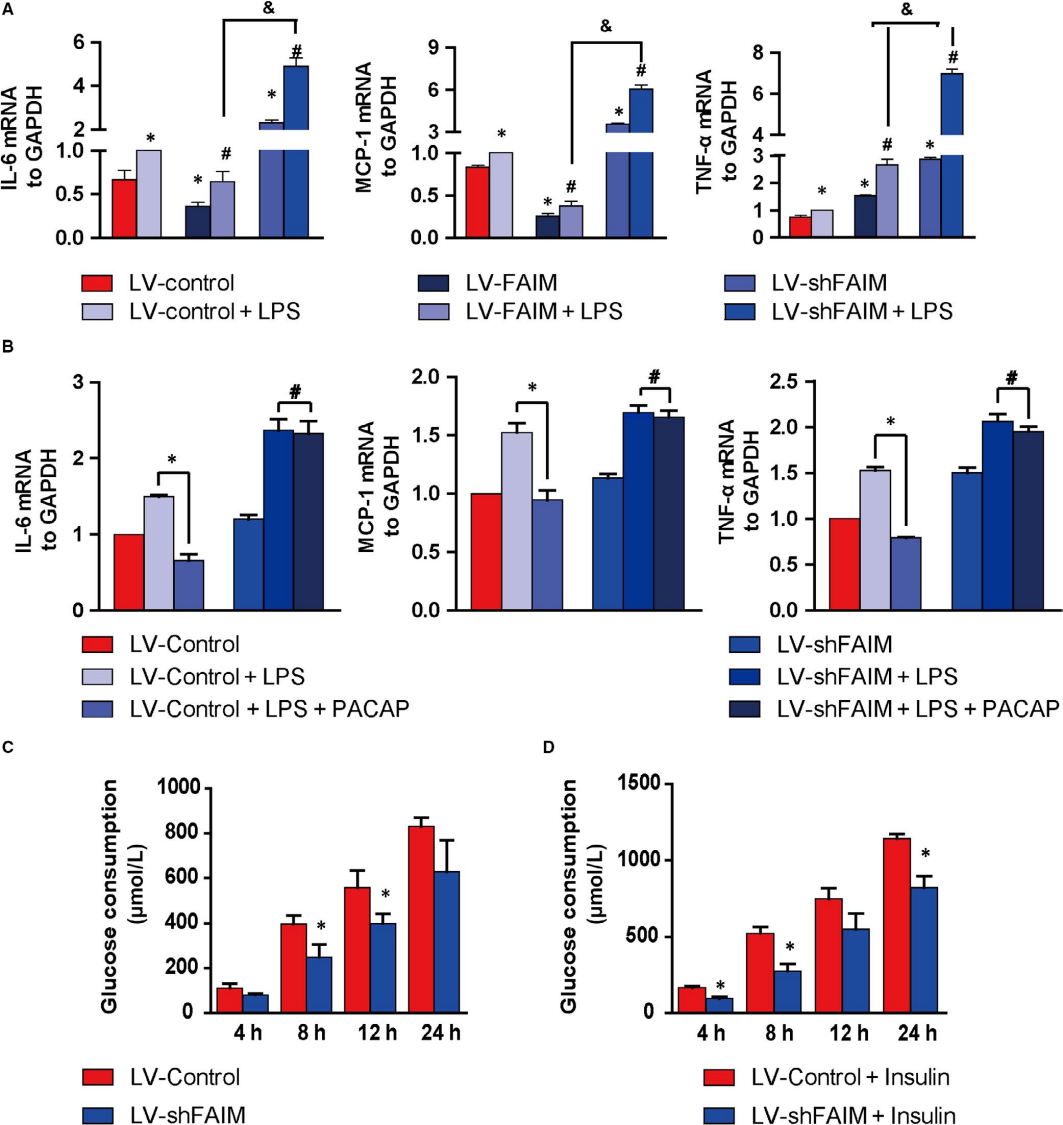
Effects of FAIM on inflammatory cytokine production and glucose metabolism. A, Cells were cultured for 24 h and then were stimulated with 0.1 g/mL LPS for 6 h. The expression levels of IL‐6, MCP‐1 and TNF‐α were analysed by real‐time PCR. *P < 0.01, groups versus LV‐Control group; #*P* < 0.01, groups versus LV‐Control + LPS group; &*P* < 0.01, LV‐shFAIM + LPS group versus LV‐FAIM + LPS group. B, PACAP was added to rescue the effects of FAIM knockdown, and then the mRNA expression levels of pro‐inflammatory factors were detected. *P < 0.01, PACAP + LPS groups versus LPS groups; #*P* > 0.05, PACAP + LPS groups versus LPS groups. C, Accumulated glucose consumption of AML12 cells. The AML12 cells were cultured in 6‐well plates, the glucose levels in the medium were detected at 4, 8, 12 and 24 h, and the cumulative glucose consumption was calculated. *P < 0.01, LV‐shFAIM groups versus LV‐Control groups. D, After cells were treated with insulin (100 nM), the cumulative glucose consumption was calculated at 4, 8, 12 and 24 h. *P < 0.01, LV‐shFAIM + insulin groups versus LV‐Control + insulin groups. Data are presented as mean ± SEM

Treated lentivirus infected AML02 cells with 0.1 g/mL of LPS for 6 hours, the expression of inflammatory factors elevated (Figure 3A). It is worth noting that FAIM overexpression suppressed IL‐6, MCP‐1 and TNF‐α, while FAIM knockdown promoted these pro‐inflammatory cytokines (Figure 3). More importantly, in the LV‐Control groups, 1 µmol/L PACAP inhibited the expression of LPS induced inflammatory factors. However, in the LV‐shFAIM groups, 1 µmol/L PACAP had no effect on the expression of inflammatory factors (Figure 3B).

In addition, FAIM knockdown decreased the cumulative glucose consumption and insulin sensitivity (Figure 3C,D). FAIM knockdown decreased the protein expression of SCD1 in AML12 cell treatment with PACAP (Figure S4C).

### 3.5 The PACAP‐FAIM pathway ameliorates liver metabolism and obesity

We utilized a high‐fat diet to induce obesity. One group of 6‐week‐old C57BL/6J wild‐type mice was fed a high‐fat diet (HFD), and another group was fed standard chow (SD) for weeks, long enough to cause obesity, then the body weight and blood glucose levels were evaluated. After 14 weeks, HFD‐fed animals presented a significant increase in the body weight (32 ± 0.9 g) and blood glucose levels (9.08 ± 0.82 mmol/L) compared with SD‐fed animals (body weight 27 ± 0.8, blood glucose 7.3 ± 0.71 mmol/L); when the mice were 16 weeks, the body weight of HFD‐fed mice increased by 25% compared with that of SD‐fed mice (Figure S6A and B). Hence, the obese mouse model was established.

Liver is the main places of energy metabolism and are closely related to the occurrence of obesity. Representative photographs of liver and adipose tissue (WAT) of the high‐fat group and normal control group comprising males at 16 weeks of age demonstrated liver and adipose hypertrophy in obesity males (Figure S6C). The levels of serum triglycerides and total cholesterol were higher in the high‐fat diet‐induced mice than in the control group (Figure S6D and E). Stearoyl‐CoA desaturase 1 (SCD‐1) is the rate‐limiting enzyme in the biosynthesis of monounsaturated fatty acids, and we extracted liver tissue to analyse the levels of FAIM and PACAP in the obesity group (n = 5) and control group (n = 5); obese mice had significantly lower levels of FAIM and PACAP, while they had significantly higher levels of SCD1 (Figure S6F).

**Figure 4:**
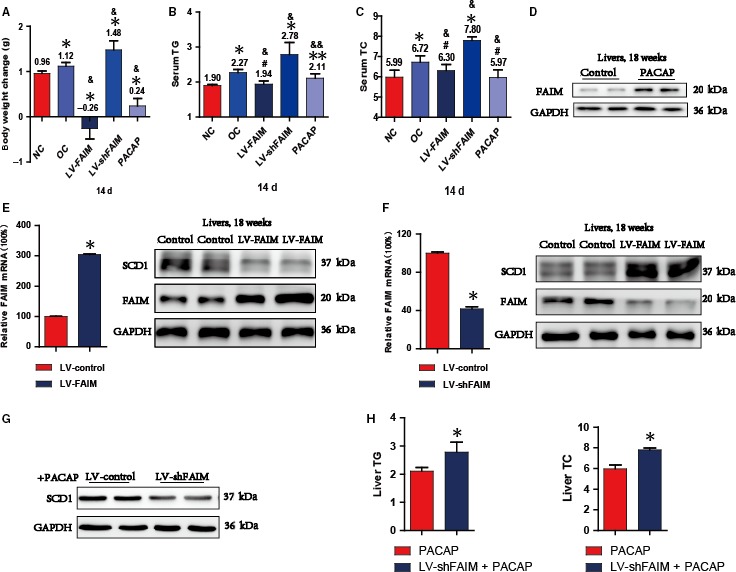
The PACAP‐FAIM pathway regulates liver metabolism and obesity A, Body weight changes of FAIM‐deficient mice (LV‐shFAIM, n = 5), FAIM‐overexpression mice (LV‐FAIM, n = 5) and mice injected with PACAP (100 nmol/L) 200 μL every other day (PACAP, n = 5) for 14 days. *P < 0.01, groups versus control groups (NC); & *P* < 0.01, groups versus obesity groups (OC). B and C, The serum triglyceride (TG) and total cholesterol (TC) levels were detected after lentivirus infection for 14 days. Two independent experiments, with five per group in each experiment. *P < 0.01, **P < 0.05 or #*P* > 0.05, groups versus control groups (NC); & *P* < 0.01 or &&*P* < 0.05, groups versus obesity groups (OC). D, The expression of FAIM was analysed in PACAP groups. E, Obese mice injected with LV‐FAIM or LV‐control were analysed for FAIM expression (left) and FAIM and SCD1 abundance (right). *P < 0.01, LV‐FAIM group versus LV‐control group. F, Obese mice injected with LV‐shFAIM or LV‐control were analysed for FAIM expression (left) and FAIM and SCD1 abundance (right). *P < 0.01, LV‐shFAIM group versus LV‐control group. G, The protein expression of SCD1 in LV‐control group cell and LV‐shFAIM group cell with treatment with 1 μmol/L PACAP. H, The TG and TC contents of each group in mouse liver tissues. *P < 0.01, PACAP group versus LV‐shFAIM + PACAP group (n = 5). Data are presented as mean ± SEM

**Figure 5:**
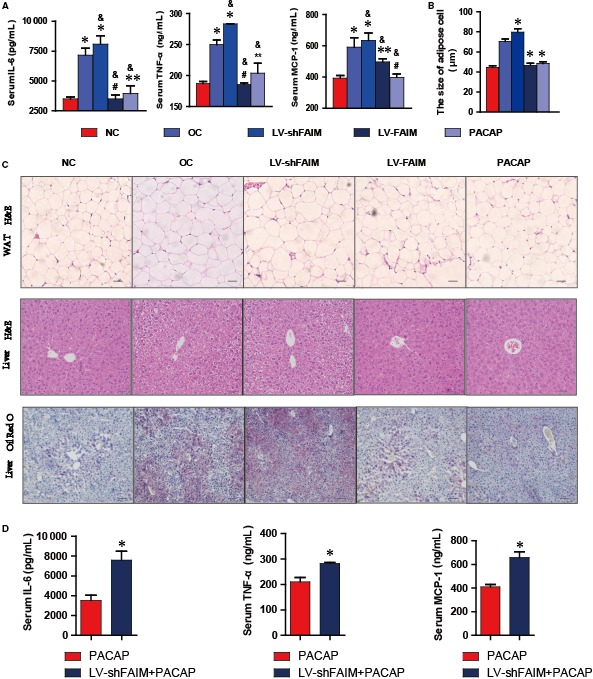
Effects of FAIM and PACAP on inflammatory factors and liver steatosis. A, Serum levels of inflammatory factors. Mice were injected with LV‐FAIM, LV‐shFAIM or PACAP. After 14 days, the levels of MCP‐1, IL‐6 and TNF‐α in the serum were detected. #*P* > 0.05, **P < 0.05 and *P < 0.01, groups versus control group (NC, n = 5); &*P* < 0.01, groups versus obesity group (OC, n = 5). B, Diameters of abdominal adipocytes in different groups. *P < 0.01, groups versus obesity control group (OC, n = 5). C, Hepatic steatosis assay using H&E (scale bars, 100 μmol/L) and Oil Red O staining (scale bars, 200 μmol/L). D, The levels of MCP‐1, IL‐6 and TNF‐α in the serum of each group mouse. *P < 0.01, PACAP group versus LVshFAIM + PACAP group (n = 5). Scale bar, 100 μmol/L. Data are presented as mean ± SEM

To explore whether the PACAP‐FAIM pathway could decrease obesity, we injected the LV‐FAIM or LV‐shFAIM into the tail vein of obese mice. Western blot results showed that the liver protein levels of FAIM were increased in the LV‐FAIM group (n = 5, Figure 4A) but were decreased in the LV‐shFAIM group (n = 5, Figure 4E). After treatment for 14 days, the mouse body weight, serum triglyceride level and total cholesterol level were increased in the LV‐shFAIM group compared with those in the obese control group (n = 5, OC) but were decreased in the LV‐FAIM group (Figure 4A4). Meanwhile, we injected PACAP into mice at 100 nmol/L in 200 µL of saline once each day for 14 days and found that FAIM expression in the liver was increased (n = 5, Figure 5E) and the levels of serum triglycerides and total cholesterol were lower than those in the obese control group (Figure 5B and 5). Focused on the change in body weight, we found that FAIM‐knockdown in obese mice significantly promoted the weight gain. However, FAIM‐ overexpression suppressed the weight gain, and injection of PACAP also rescued the abnormal obese weight gain (Figure 4D).

Next, we investigated whether SCD1 was involved in mediating FAIM's effect on adipogenesis in the liver. The LV‐FAIM and LV‐shFAIM lentiviruses were injected into obese mice, and the abundance of hepatic SCD1 protein was significantly decreased by FAIM overexpression and increased by FAIM knockdown (Figure 4E and 4). PACAP treatment decreased the protein expression of SCD1 in the FAIM‐knockdown AML12 cell (Figure 4G), and injected PACAP at a concentration of 100 nmol/L in 200 µL of saline into mice every other day in LV‐shFAIM group mice for 14 days, and compared with the PACAP group, the hepatic contents of TG, TC were analysed, the TG and TC contents of PACAP group were significantly lower than the LV‐shFAIM + PACAP group (Figure 4H).

Therefore, it was conceivable that the PACAP‐FAIM pathway affected hepatic metabolism, reducing the fat abundance.

### 3.6 The PACAP‐FAIM pathway decreases inflammatory and adipose infiltration in the liver

Obesity and chronic inflammation are closely related, and increasing attention has been given to the effect of inflammatory cytokines on the metabolism in obese patients. In rodents, anti‐inflammatory treatments have been demonstrated to dampen inflammation‐driven insulin resistance.

After lentivirus or PACAP treatment for 14 days, we collected mouse plasma samples. Next, we analysed the levels of the inflammatory factors in each group. We found that the levels of MCP‐1, IL‐6 and TNF‐α in obese mice (Obesity) were 1.51, 1.75 and 1.43‐fold higher than those in normal control mice (Control), respectively (Figure 5A). The serum MCP‐1 level of the FAIM‐overexpression group (LV‐FAIM) and PACAP‐injected group (PACAP) was dramatically declined compared with that in the obesity group (Obesity), while the serum MCP‐1 level of the FAIM‐knockdown group (LV‐shFAIM) rose continuously compared with that in the obesity group (Obesity) (Figure 5A). In addition, the serum IL‐6 and TNF‐α levels of the FAIM‐overexpression group (LV‐FAIM) and PACAP‐injected groups (PACAP) were lower than those of the obesity group (Obesity), but the serum IL‐6 and TNF‐α levels of the FAIM knockdown group (LV‐shFAIM) were also increased compared with those of the obesity group (Obesity) (Figure 5A).

To gain insight into the cause of obesity in mice, we examine their fat tissue. The size of the epididymal adipocytes pad in the four groups (OC, LV‐shFAIM, LV‐FAIM, PACAP) of obese mice was markedly enlarged compared with that of normal control mice. Knockdown of FAIM with LV‐shFAIM significantly enlarged the diameter of adipocytes, while overexpression of FAIM and PACAP treatment decreased the adipocyte diameter (Figure 5B,C upper). Liver steatosis was observed in the OC and LV‐shFAIM groups from H&E staining, and the overexpression of FAIM and PACAP could modulate obese‐caused liver steatosis (Figure 5C middle). Similarly, Oil Red O staining showed adipose infiltration in the OC and LV‐shFAIM groups and moderate infiltration in the LV‐FAIM and PACAP groups (Figure 5C down). FAIM‐knockdown affects PACAP anti‐inflammatory response (Figure 5D). Thus, the **PACAP‐FAIM pathway** is important regulators of inflammatory and adipose infiltration in the liver.

### 3.7 FAIM down‐regulates SREBP signalling and restrains lipogenesis in mouse liver tissues

The key genes of lipid metabolism in the liver were examined to reveal the mechanism of the PACAP‐FAIM pathway in lipogenesis. Genes encoding enzymes involved in lipogenesis include fatty acid synthase (FAS), SCD‐1, sterol regulatory element binding protein (SREBP) and 3‐hydroxy‐3‐methyl‐glutaryl‐CoA reductase (HMGCR). FAIM could enhance the activation of SREBP signalling, as well as that of downstream lipogenic target genes such as FAS and SCD1.

**Figure 6:**
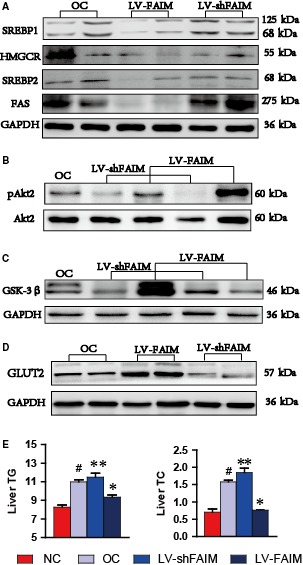
FAIM‐regulated SREBP and Akt2 signalling enhance hepatic cell glucose uptake. A, Expression of SREBP 1, FAS, SREBP 2 and HMGCR in OC, LV‐shFAIM and LV‐FAIM group of liver tissues. *P < 0.01, groups versus obesity group. B, Akt2 phosphorylation and total Akt2 were analysed in the livers of obese mice after injection with LV‐FAIM or LV‐shFAIM for 14 days. C, The protein level of GSK‐3β was analysed in the livers of obese mice after injection with LV‐FAIM or LV‐shFAIM for 14 days. D, The protein level of GLUT2 was analysed in the livers of obese mice after injection with LV‐FAIM or LV‐shFAIM for 14 days. E, The TG and TC contents of each group in mouse liver tissues. #*P* < 0.01, obesity group versus control group; *P < 0.01 or **P < 0.05, groups versus obesity group. Data are presented as mean ± SEM

In LV‐FAIM mouse liver, the protein expression of SREBP1 was markedly increased, while that in LV‐shFAIM mice was reduced. Hence, the downstream protein FAS followed the same pattern (Figure 6A). These findings reinforced that FAIM overexpression reduced SREBP1, FAS and SCD1 expression. We also observed up‐regulation of the SREBP‐2 pathway, which preferentially activates cholesterol synthesis in the liver by targeting HMGCR. In the LV‐FAIM mouse groups, the levels of SREBP‐2 and HMGCR were lower than those in the obese control mice; however, in LV‐shFAIM mice, the levels of SREBP‐2 and HMGCR were higher than that those in obese control mice (Figure 6A).

Besides, we analysed the TG and TC contents of liver tissue in each group and found that the TG and TC contents of the LV‐FAIM mouse groups were decreased compared with those in the obese control mice (Figure 6E). These data showed that the PACAP‐FAIM pathway down‐regulates SREBP signalling and restrains lipogenesis in mouse liver tissue.

### 3.8 FAIM enhances glucose uptake in hepatic cells

Hepatic insulin resistance is an important pathophysiological feature of obesity, and PI3K/Akt2 are the main components of the insulin signalling pathway. In addition, previous studies have shown that FAIM expression is essential for Akt2 activation. In AML12 cells, we proved that FAIM deficiency led to impaired insulin sensitivity (Figure 3D). Next, we isolated hepatocytes from lentivirus‐transfected mice and found that Akt2 phosphorylation (Ser474) was reduced in the LV‐shFAIM group, but was increased in the liver in the LV‐FAIM group, compared with that in the obese control mice (Figure 6B).

Glycogen synthase kinase‐3β (GSK‐3β) and GLUT2, key enzymes of glycogen metabolism, are controlled by the insulin signalling pathway and are involved in glycogen metabolism. We found that the protein levels of GSK‐3β and GLUT2 were significantly higher in LV‐FAIM mice, but were significantly lower in LV‐shFAIM mice, than in obese control mice (Figure 6C and 6). These findings reinforced that FAIM could enhance insulin signalling and glucose uptake, indicating that FAIM can regulate hepatic cell glycogen synthesis.

Finally, a graphic abstract is shown.

## 4 DISCUSSION

Obesity is associated with metabolic diseases such as type 2 diabetes and hepatosteatosis.^19^ The condition is characterized by insulin resistance, high blood sugar, dyslipidaemia and chronic inflammation.^20^ PACAP plays an important role in the metabolism regulation, and is expressed in many tissues.^21^ In this study, we found the serum levels of FAIM was consistent with the PACAP, but opposite to the pro‐inflammatory factors and lipid parameters. These observations are consistent with our earlier studies showing that FAIM might be a glucose and lipid metabolism regulator.^18^

In order to study the relationship of PACAP and FAIM, we chose the human normal hepatocyte (L02 cell line) and found that PACAP elevated the protein of FAIM. Moreover, there are three receptors of PACAP, VPAC1, VPAC2 and PAC1, and many reports showed that PACAP worked mainly though PAC1 in neural and reproductive tissues.^11^ Although the three different receptors of PACAP were all expressed in L02 cells, our data revealed that PACAP up‐regulated FAIM only through the PAC1 receptor. When we added selective inhibitors of the other two receptors of PACAP, the expression of FAIM unchanged.

Obesity is often associated with insulin resistance and chronic systemic inflammation, the blood inflammatory markers, including monocyte chemoattractant protein‐1 (MCP‐1), RANTES, IL‐6, and TNF‐α were evaluated in obese human than normal controls.^22^, ^23^ To explore the effects of FAIM on the inflammatory and insulin sensitivity, we overexpressed FAIM in obese mice with lentivirus, found that the levels of inflammatory factors, blood glucose, serum triglycerides and total cholesterol decreased. Studies about FAIM mainly focused on the neural and reproductive organ development, only few reports showed that the FAIM2, another member of FAIM family, was associated with obesity and dyslipidaemia. From our data, we proved the novel role of FAIM was as a regulator of the anti‐inflammatory and metabolism protein in obesity.

Liver is the key organ for energy metabolism, excess glucose and lipids will cause fatty liver and steatosis. Lipogenesis marker genes include FAS, SCD‐1, SREBP and HMGCR are important metabolic enzymes in the liver.^24^, ^25^, ^26^, ^27^ After we knocked down the FAIM with LV‐shFAIM, the levels of these lipogenesis enzymes elevated, but declined in the LV‐FAIM mouse liver tissue. On the other hand, FAIM may down‐regulate SREBP signalling and restrain lipogenesis in mouse liver.

AKT is the main component of the insulin signalling pathway, and earlier studies had shown that FAIM expression is essential for the activation of Akt2 in thymocytes and myeloma cells.^28^ Our current study found that Akt2 phosphorylation (Ser474) was increased in the liver of LV‐FAIM mice, while reduced in LV‐shFAIM mice compared with that in obese control mice. Undoubtedly, the expression levels of key enzymes of glycogen metabolism (GLUT2 and GSK‐3β) followed the same pattern as Akt2.

In summary, PACAP alleviate inflammation and steatosis in the liver, ameliorated glucose and lipid metabolism in obesity via up‐regulating FAIM. These findings provided a novel pathway and therapy target for the prevention and treatment of obesity, chronic inflammatory, insulin resistance and related metabolic disorders.

## CONFLICT OF INTEREST

All the authors declare that they had no conflicts of interest.

## AUTHORS’ CONTRIBUTIONS

Xing Xiao, Pei Qiu, Sun Yan, An Hong, Yi Ma performed contributing to the conception and design; Xing Xiao, Pei Qiu, Hui‐zhen Gong, Xue‐ming Chen involved in analysing and interpreting data; Xing Xiao performed drafting the article; Xing Xiao, Pei Qiu, Yi Ma performed revising it critically for important intellectual content. All authors involved in approving the final version to be published.

## References

[1] Null R O I C, Consultation W . Obesity: preventing and managing the global epidemic.[J]. Report of A Who Consultation on Obesity. 2000; 15(1):18‐30. 11234459

[2] Sonnenburg JL , Bäckhed F . Diet‐microbiota interactions as moderators of human metabolism. Nature. 2016;535:56. 27383980 10.1038/nature18846 PMC5991619

[3] Gomes MB , Giannella Neto D , Mendonça ED et al. Nationwide multicenter study on the prevalence of overweight and obesity in type 2 diabetes mellitus in the Brazilian population. Arq Bras Endocrinol Metabol. 2006;50:136‐144. 16628286 10.1590/s0004-27302006000100019

[4] Gaal L , Mertens IL , Block C . Mechanisms linking obesity with cardiovascular disease. Nature. 2006;444:875‐880. 17167476 10.1038/nature05487

[5] Gregor MF , Hotamisligil GS . Inflammatory mechanisms in obesity. Annu Rev Immunol. 2011;29:415. 21219177 10.1146/annurev-immunol-031210-101322

[6] Singer G , Granger N . Inflammatory responses underlying the microvascular dysfunction associated with obesity and insulin resistance. Microcirculation. 2007;14:375‐387. 17613809 10.1080/10739680701283158

[7] Dominguez H , Storgaard H , Rask‐Madsen C , et al. Metabolic and vascular effects of tumor necrosis factor‐α blockade with etanercept in obese patients with type 2 diabetes. J Vasc Res. 2005;42:517. 16155368 10.1159/000088261

[8] Stanley TL , Zanni MV , Johnsen S , et al. TNF‐alpha antagonism with etanercept decreases glucose and increases the proportion of high molecular weight adiponectin in obese subjects with features of the metabolic syndrome. J Clin Endocrinol Metab. 2011;96:146‐150. 10.1210/jc.2010-1170 PMC3038481 21047923

[9] Figiel M , Engele J . PACAP, a neuron‐derived peptide regulating glial glutamate transport and metabolism. J Neurosci. 2000;20:3596‐3605. 10804201 10.1523/JNEUROSCI.20-10-03596.2000 PMC6772687

[10] Hashimoto H , Nogi H , Mori K , et al. Distribution of the mRNA for a pituitary adenylate cyclase‐activating polypeptide receptor in the rat brain: an in situ hybridization study. J Comp Neurol. 1996;371:567‐577. 8841910 10.1002/(SICI)1096-9861(19960805)371:4<567::AID-CNE6>3.0.CO;2-2

[11] Vu JP , Goyal D , Luong L , et al. PACAP intraperitoneal treatment suppresses appetite and food intake via PAC1 receptor in mice by inhibiting ghrelin and increasing GLP‐1 and leptin. Am J Physiol Gastrointest Liver Physiol. 2015;309:G816. 26336928 10.1152/ajpgi.00190.2015 PMC4652141

[12] Sakamoto K , Kuno K , Takemoto M , et al. Pituitary adenylate cyclase‐activating polypeptide protects glomerular podocytes from inflammatory injuries. J Diabetes Res. 2015;2015:727152. 25821833 10.1155/2015/727152 PMC4363873

[13] Cho H , Mu J , Kim JK , et al. Insulin resistance and a diabetes mellitus‐like syndrome in mice lacking the protein kinase Akt2 (PKB beta). Science. 2001;292:1728. 11387480 10.1126/science.292.5522.1728

[14] Huo J , Xu S , Lam KP . Fas apoptosis inhibitory molecule regulates T cell receptor‐mediated apoptosis of thymocytes by modulating Akt activation and Nur77 expression. J Biol Chem. 2010;285:11827‐11835. 20178987 10.1074/jbc.M109.072744 PMC2852919

[15] Schneider TJ , Fischer GM , Donohoe TJ , Colarusso TP , Rothstein TL . A novel gene coding for a Fas apoptosis inhibitory molecule (FAIM) isolated from inducibly Fas‐resistant B lymphocytes. J Exp Med. 1999;189:949. 10075978 10.1084/jem.189.6.949 PMC2193037

[16] Rothstein TL , Zhong X , Schram BR , et al. Receptor‐specific regulation of B‐cell susceptibility to Fas‐mediated apoptosis and a novel Fas apoptosis inhibitory molecule. Immunol Rev. 2000;176:116‐133. 11043772 10.1034/j.1600-065x.2000.00616.x

[17] Du K , Herzig S , Kulkarni RN , Montminy M . TRB3: a tribbles homolog that inhibits Akt/PKB activation by insulin in liver. Science. 2003;300:1574‐1577. 12791994 10.1126/science.1079817

[18] Huo J , Ma Y , Liu J‐J , et al. Loss of Fas apoptosis inhibitory molecule leads to spontaneous obesity and hepatosteatosis. Cell Death Dis. 2016;7:e2091. 26866272 10.1038/cddis.2016.12 PMC4849152

[19] Kolotkin RL , Meter K , Williams GR . Quality of life and obesity. Obesity reviews. 2001;2(4):219-229. 12119993 10.1046/j.1467-789x.2001.00040.x

[20] Boden G , Sargrad K , Homko C , Mozzoli M , Stein TP . Effect of a low‐carbohydrate diet on appetite, blood glucose levels, and insulin resistance in obese patients with type 2 diabetes. Ann Intern Med. 2005;142:403‐411. 15767618 10.7326/0003-4819-142-6-200503150-00006

[21] Reglodi D , Kiss P , Lubics A , Tamas A . Review on the protective effects of PACAP in models of neurodegenerative diseases in vitro and in vivo. Curr Pharm Des. 2011;17:962. 21524257 10.2174/138161211795589355

[22] Dandona P , Aljada A , Chaudhuri A , Mohanty P , Garg R . Metabolic syndrome A comprehensive perspective based on interactions between obesity, diabetes, and inflammation. Circulation. 2005;111:1448‐1454. 15781756 10.1161/01.CIR.0000158483.13093.9D

[23] Shoelson SE , Herrero L , Naaz A . Obesity, inflammation, and insulin resistance. Gastroenterology. 2014;132:2169‐2180. 10.1053/j.gastro.2007.03.059 17498510

[24] Choi K‐M , Jeon YS , Kim W , et al. Xanthigen attenuates high‐fat diet‐induced obesity through down‐regulation of PPARγ and activation of the AMPK pathway. Food Sci Biotech. 2014;23:931‐935.

[25] Mauvoisin D , Rocque G , Arfa O , Radenne A , Boissier P , Mounier C . Role of the PI3‐kinase/mTor pathway in the regulation of the stearoyl CoA desaturase (SCD1) gene expression by insulin in liver. J Cell Commun Signal. 2007;1:113‐125. 18481202 10.1007/s12079-007-0011-1 PMC2275876

[26] Ranganathan G , Unal R , Pokrovskaya I , et al. The lipogenic enzymes DGAT1, FAS, and LPL in adipose tissue: effects of obesity, insulin resistance, and TZD treatment. J Lipid Res. 2006;47:2444‐2450. 16894240 10.1194/jlr.M600248-JLR200 PMC1850099

[27] Yahagi N , Shimano H , Hasty AH , et al. Absence of sterol regulatory element‐binding protein‐1 (SREBP‐1) ameliorates fatty livers but not obesity or insulin resistance in Lep(ob)/Lep(ob) mice. J Biol Chem. 2002;277:19353‐19357. 11923308 10.1074/jbc.M201584200

[28] Vareda P , Saldanha LL , Camaforte N , et al. Myrcia bella leaf extract presents hypoglycemic activity via PI3k/Akt insulin signaling pathway. Evid Based Complementary Altern Med. Medicine. 2014:1‐11. 10.1155/2014/543606 PMC4020406 24872834

