# Supplementary figures and images for: PACAP ameliorates hepatic metabolism and inflammation through up‐regulating FAIM in obesity
Source: J Cell Mol Med. 2019 Jul 3;23(9):5970–80. doi: 10.1111/jcmm.14453 (PMC6714231; doi:10.1111/jcmm.14453)

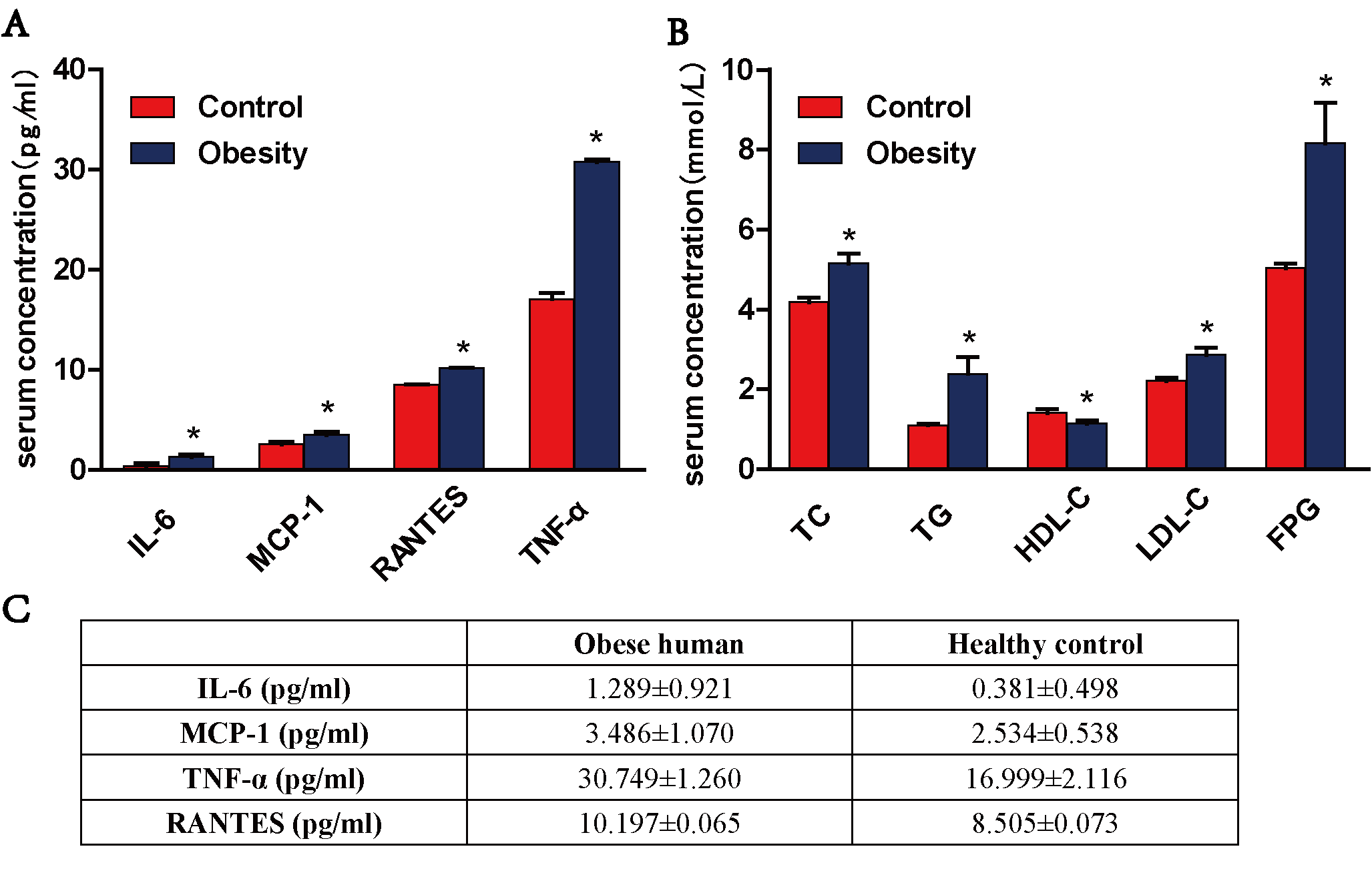

Supplement: Supplementary file 1 [file JCMM-23-5970-s001.tif]

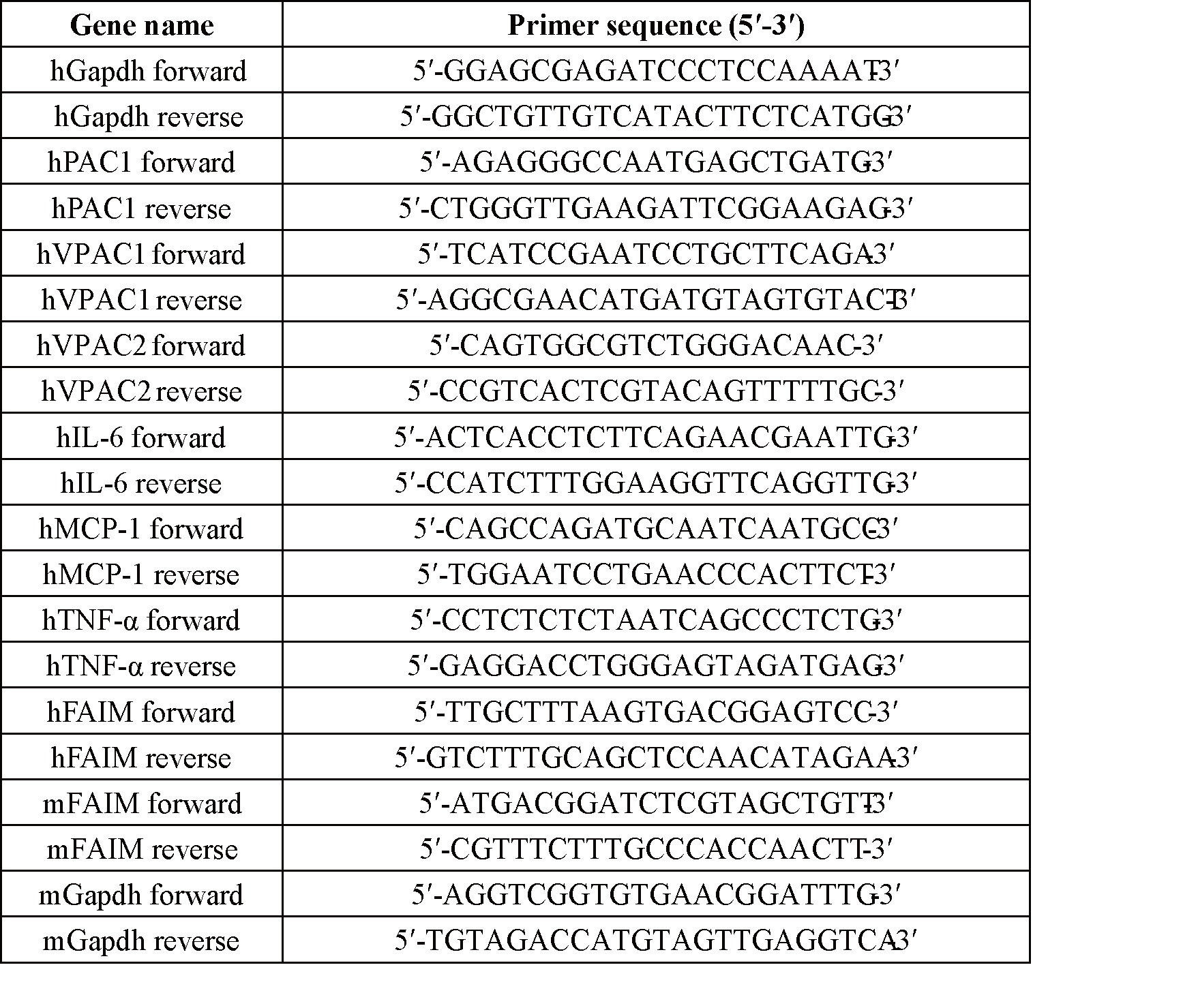

Supplement: Supplementary file 2 [file JCMM-23-5970-s002.tif]

**FIGURE S2** The relative PCR primer sequences.
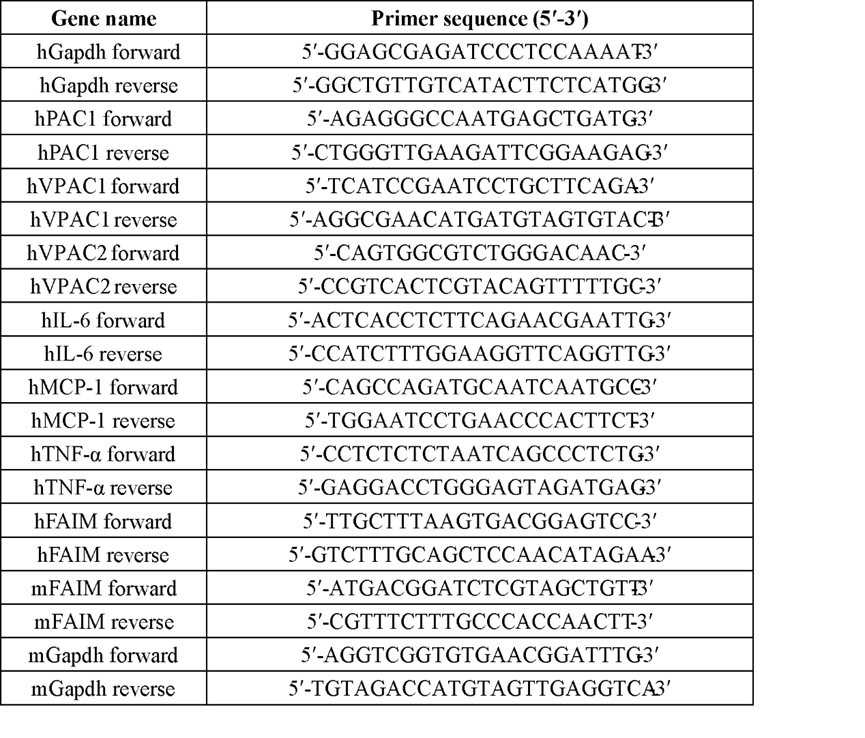

Supplement: Supplementary file 3 [file JCMM-23-5970-s003.docx]

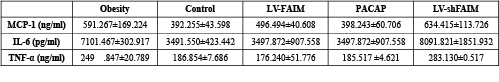

Supplement: Supplementary file 4 [file JCMM-23-5970-s004.tif]

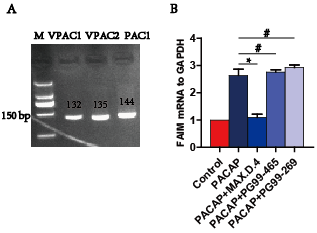

Supplement: Supplementary file 5 [file JCMM-23-5970-s005.tif]

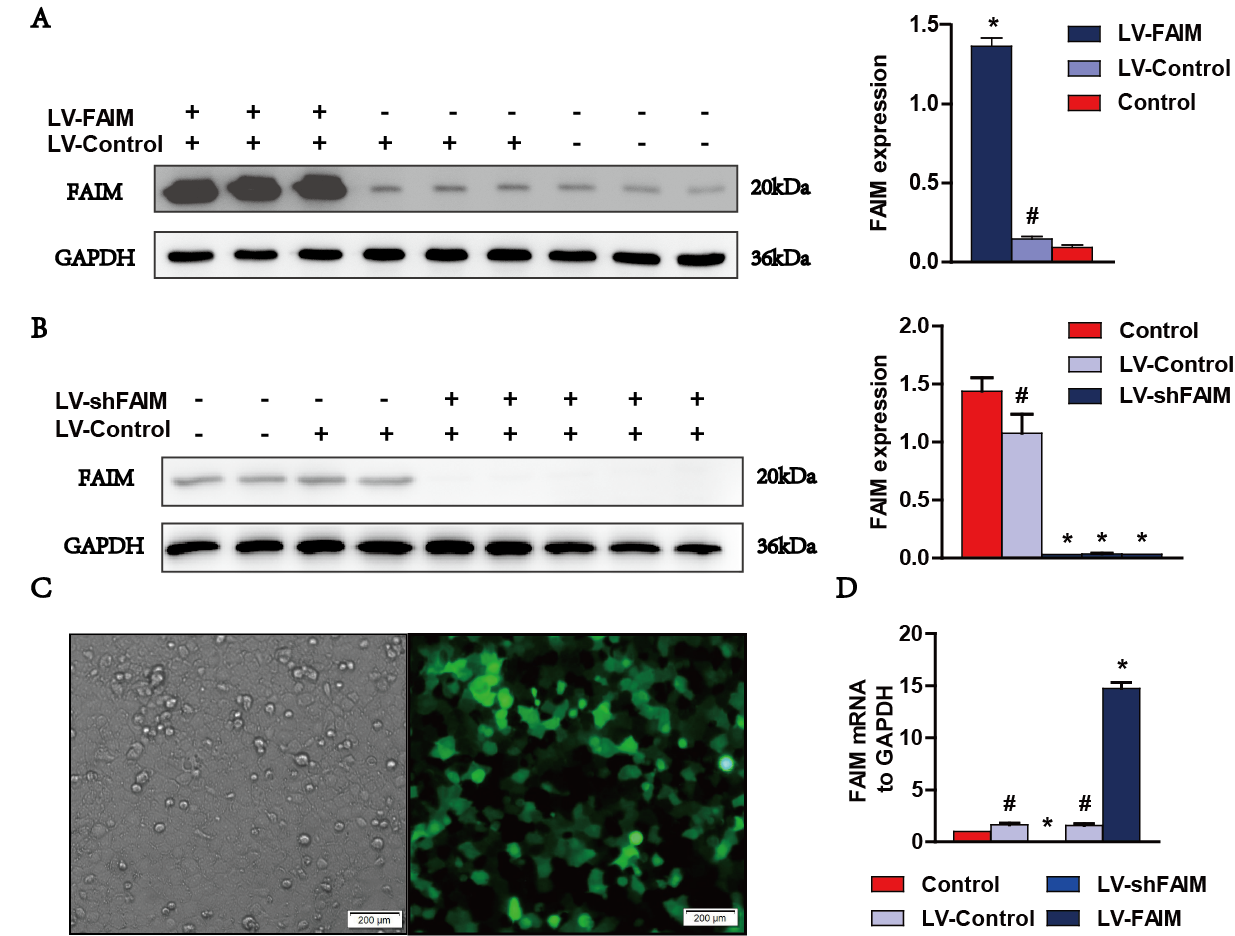

Supplement: Supplementary file 6 [file JCMM-23-5970-s006.tif]

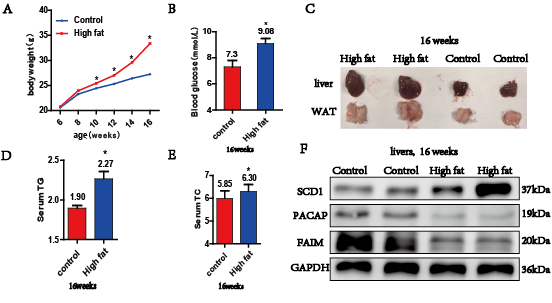

Supplement: Supplementary file 7 [file JCMM-23-5970-s007.tif]

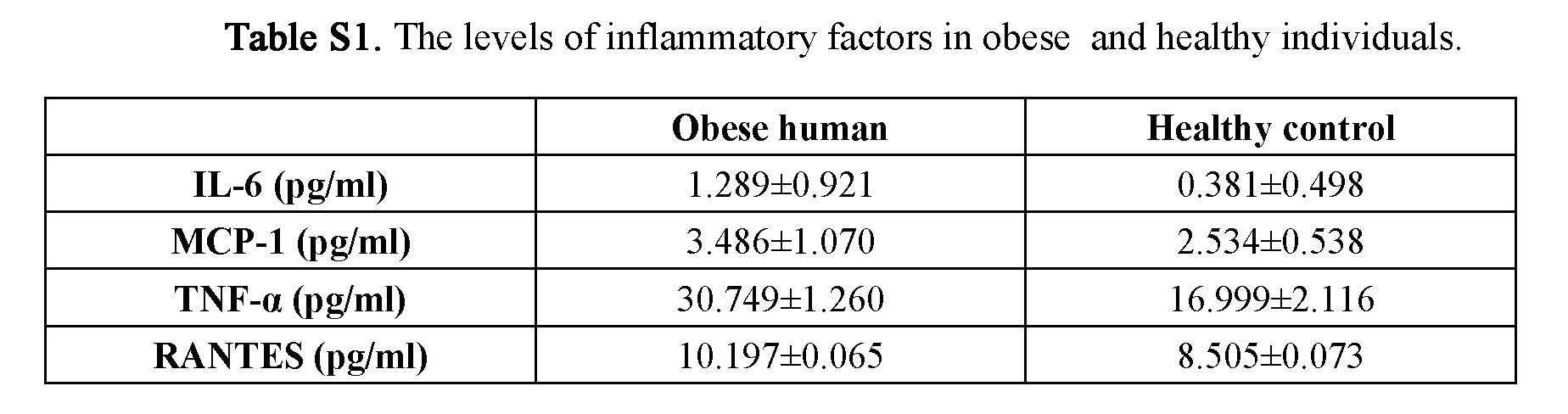

Supplement: Supplementary file 8 [file JCMM-23-5970-s008.tif]
